# Contrast Enhancement of Aneurysm Sac Post-Pipeline Treatment Interpreted as Recanalization

**DOI:** 10.7759/cureus.1732

**Published:** 2017-09-29

**Authors:** Annie J Tsay, Sara Langan, Scott Simon

**Affiliations:** 1 Medical Student, Penn State University College of Medicine; 2 Department of Neurosurgery, Penn State Hershey Medical Center

**Keywords:** flow diversion, mra, brain aneurysm, pipeline embolization device, cta

## Abstract

Multiple imaging modalities are available to evaluate aneurysms post-flow diverter (FD) placement. Though digital subtraction angiography (DSA) is the gold standard imaging modality post-FD placement, it is not perfect, and neither are other techniques, including contrast-enhanced magnetic resonance angiography (CE-MRA) and magnetic resonance imaging (MRI). We present a case of a 73-year-old woman with a right internal carotid artery (ICA) aneurysm treated with a pipeline embolization device (PED). Initial follow-up post-PED placement by three-dimensional time-of-flight (3D-TOF) MRA demonstrated aneurysm occlusion, which was confirmed by computed tomography angiography (CTA) and CE-MRA in subsequent follow-up appointments. However, repeat CE-MRA two years later suggested recanalization of the aneurysm. After discussion with neuroradiologists and follow-up with a dynamic MRA, this finding was determined to be false. These findings shed light on the potential pitfall of using CE-MRA alone or any single imaging modality in the assessment of aneurysms post-PED placement. Our case report explores various imaging modalities used in the assessment of aneurysms post-PED placement and highlights the need to use multiple techniques for an accurate assessment.

## Introduction

Flow diversion follow-up should not show the filling of an aneurysm. However, digital subtraction angiography (DSA) is an invasive procedure and cannot detect the resolution of an aneurysm. Therefore, contrast-enhanced magnetic resonance angiography (CE-MRA) ought to be the ideal imaging modality to obtain information regarding an aneurysm, vessel lumen, and the process of aneurysm resolution. We present an unusual case in which CE-MRA following pipeline embolization device (PED) placement suggested the recanalization of the aneurysm; however, upon reassessment, this result was determined to be false. This case suggests that post-PED placement, a follow-up may require multimodality imaging techniques in order to appropriately assess aneurysm resolution.

## Case presentation

**Figure 1:**
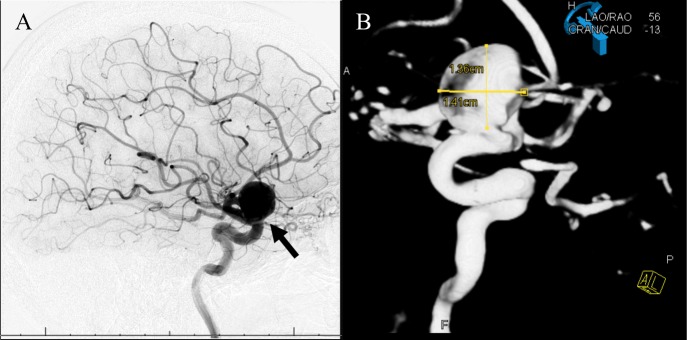
DSA of right internal carotid artery/right ophthalmic artery origin aneurysm pre-pipeline embolization device placement A) Sagittal view of spherical shaped aneurysm (solid arrow); B) Aneurysm measured to 14.1 x 13.6 mm DSA: digital subtraction angiogram

Our patient is a 73-year-old Caucasian female who presented to her primary care physician with the chief complaint of having difficulty with vision in her right eye. An ophthalmologist evaluated her and requested magnetic resonance imaging (MRI), which revealed a possible right ophthalmic artery aneurysm. She was referred to neurosurgery for further evaluation and management. Indeed, initial evaluation by DSA confirmed the presence of a spherically shaped 14.1 mm x 13.6 mm aneurysm located on the right internal carotid artery (ICA) at the origin of the right ophthalmic artery (Figure 1). The patient was counseled about treatment with a PED, along with the risks associated with the procedure.

Our patient was placed under general anesthesia in the angiography suite. A 7-French sheath (Cook Medical, Bloomington, IN) was inserted into the right femoral artery and was advanced into the aortic arch over a 5-French vertebral catheter (Cook Medical, Bloomington, IN), and a 0.035-inch guide wire where contrast was injected. Next, we used a Marksman microcatheter (Medtronic Neurovascular, Irvine, CA) placed over a Synchro microwire (Stryker, Kalamazoo, MI) to select the right middle cerebral artery (MCA). A single 5 mm x 18 mm PED was initially advanced into the right MCA, subsequently moved retrograde into the ICA just proximal to the posterior communicating (PCOM) artery, and deployed around the curvature of the cavernous carotid. A DSA follow-up run at the conclusion of the case showed the appropriate placement of the PED, adequate filling through the device, flow stasis into the aneurysm, and no occlusion of the apparent artery or branch vessels. There were no complications to report and the patient awoke from the procedure without neurologic deficits.

**Figure 2:**
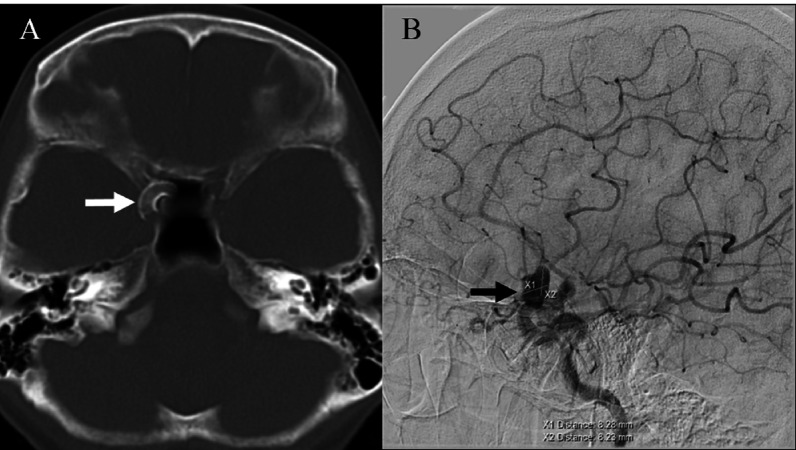
Six-month follow-up post-PED placement showed persistent filling of right ophthalmic artery aneurysm filling A) CT post-pipeline embolization device (PED) placement (solid white arrow); B) Aneurysm size reduced by 50%, measuring 8.28 x 8.23 mm (solid arrow) CT: computed tomography

At her six-month follow-up, DSA demonstrated a persistent filling of the aneurysm but with a 50% reduction in size, measuring 8.28 x 8.23 mm (Figure 2).

**Figure 3:**
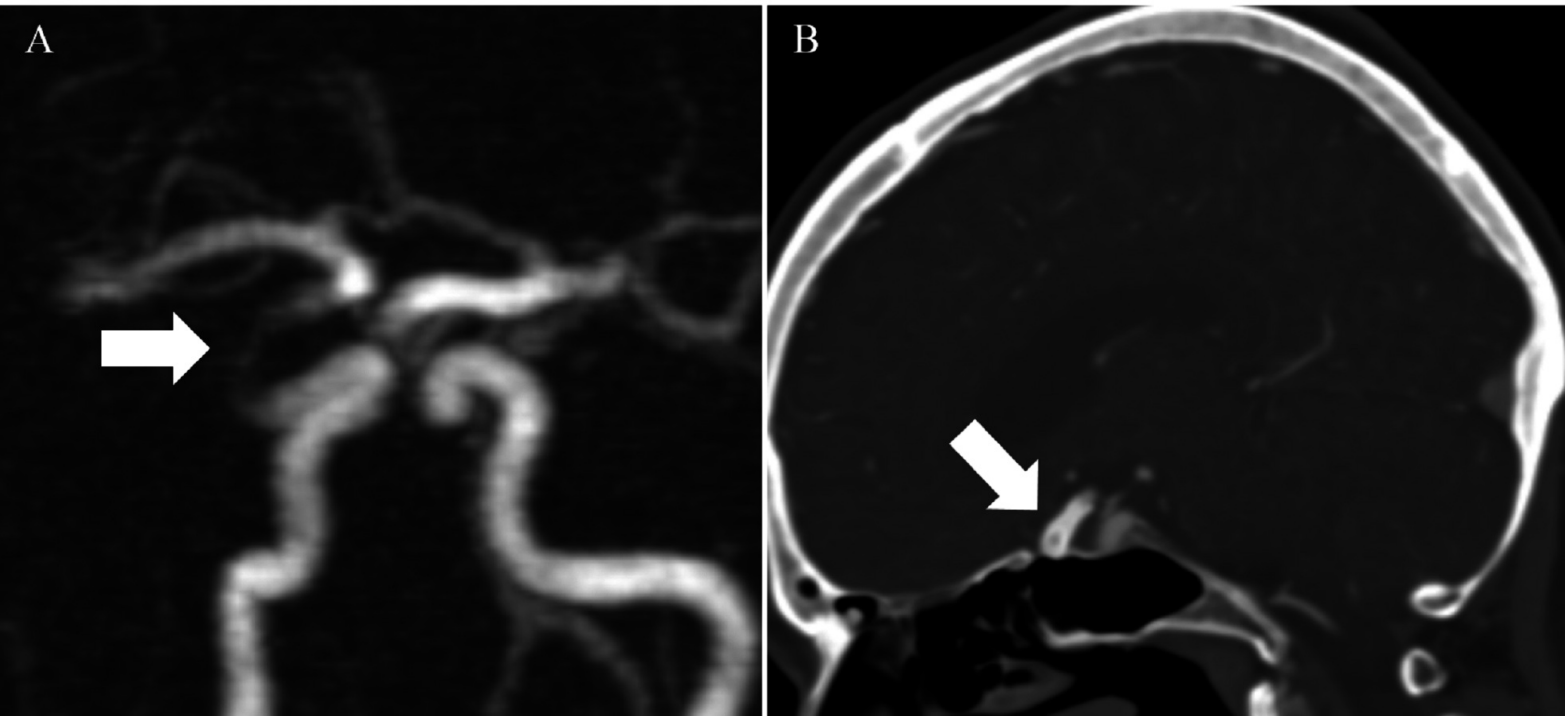
One-year follow-up post-PED placement imaging studies with 3D-TOF-MRA followed by confirmatory CTA imaging study. The 3D-TOF MRA without contrast shows no residual flow or aneurysm in right ophthalmic artery A) Three-dimensional time-of-flight magnetic resonance angiography (3D-TOF-MRA) without contrast shows no aneurysm; B) Computed tomography angiography (CTA) confirms no contrast outside of pipeline device PED: pipeline embolization device

One-year follow-up post-PED placement using three-dimensional time-of-flight (3D-TOF) MRA without contrast indicated complete occlusion without residual flow or a residual aneurysm (Figure 3).

**Figure 4:**
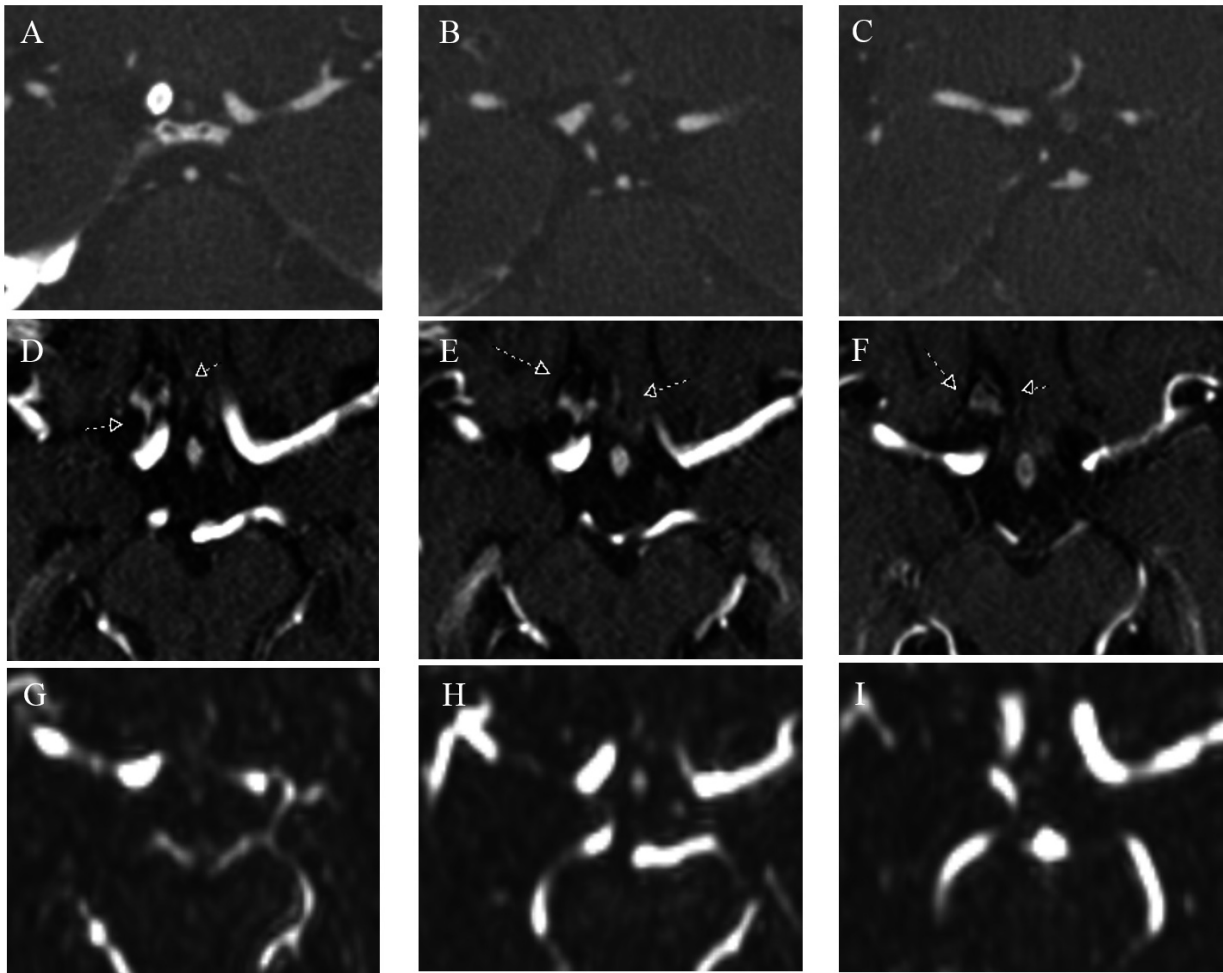
Follow-up scans in the axial plane showing the same three slices of the carotid terminus and gyrus rectus A-C) Computed tomography angiography (CTA) performed at 18 months with pipeline (bright white circle in A) and no sign of contrast filling of the previously seen aneurysm; D-F) Contrast-enhanced magnetic resonance imaging (CE-MRI) through the same anatomic location showing enhancement around the previously seen aneurysm (small arrows); G-I) Dynamic magnetic resonance angiography (MRA) confirms no flow through previously seen aneurysm, leading to the conclusion that the enhancement represented a thrombosed aneurysm wall and not recanalization

A computed tomography angiography (CTA) scan obtained at 18 months revealed no sign of aneurysm filling (Figure 4A-4C). The patient’s two-year contrast-enhanced magnetic resonance angiogram (CE-MRA) identified possible residual or recurrent filling of the coiled aneurysm sac (Figure 4D-4E). The patient refused another catheter study. Further discussion with neuroradiology led to the conclusion that enhancement of the aneurysm might be due to an occluded aneurysm wall enhancement rather than a recanalization of the aneurysm, and a dynamic MRA was suggested. This confirmed no flow (Figure 4G-4I).

## Discussion

With respect to cerebral aneurysm imaging, CTA, MRA, and DSA are the most commonly used techniques. CTA is a reliable tool to detect cerebral aneurysms, but studies using CT scanner slices ranging from 16- to 320-slice parameters have demonstrated mixed results, mostly reflecting a reduction in sensitivity when used to detect aneurysms less than 3 mm in size [1-3]. On the other hand, aneurysms larger than 4 mm in size reached near-perfect, 100% sensitivity using CTA compared to DSA [1, 4-5]. Additionally, CTA, like DSA, requires contrast administration, while MRI/MRA techniques generally do not require contrast agents.

Noncontrast requiring imaging techniques are advantageous, as they eliminate the possibility of an adverse event caused by an allergic reaction or an underlying renal condition. Among MRA technologies, studies have shown that CE-MRA and time-of-flight (TOF)-MRA are comparable in terms of sensitivity to detect cerebral aneurysms. A key difference between the two techniques is that a TOF-MRA is a dynamic imaging modality used to visualize flow within vessels without the use of contrast dye, while CE-MRA requires contrast dye injection. Though MRA may seem superior to CTA in many respects, it has poorer spatial resolution and is, in turn, not the preferred technique for diagnostic or for an acute setting evaluation of aneurysms. MRA is more commonly used for follow-up or for screening tests to avoid the successive administration of contrast [1]. To date, no single imaging technique provides absolute precision for identifying cerebral aneurysms.

The protocol for assessing aneurysms post-flow diverter (FD) placement remains controversial. Some argue that TOF-MRA offers higher quality images of small branch vessels as compared to CTA, possibly due to the lack of contrast contamination [1], while others advocate for CE-MRA over TOF-MRA in follow-up assessments post-FD placement. A prospective single-center study performed from January 2009 to January 2013 (n = 23 aneurysms) by Attali, et al. concluded that CE-MRA was better than 3D-TOF-MRA in determining aneurysm occlusion and parent artery patency post-FD placement. Interobserver agreement using simplified scales for occlusion (Montreal) and intermodality agreement for aneurysm occlusion and parent artery patency were higher for CE-MRA compared to 3D-TOF-MRA [6].

Similarly, Boddu, et al. demonstrated that CE-time-resolved MRA (CE-TR-MRA) is more reliable than 3D-TOF-MRA for follow-up of intracranial aneurysms following PED placement in a 24-month period study (n = 37 aneurysms). CE-TR-MRA provided better visualization than 3D-TOF-MRA and more accurately reflected DSA findings [7]. However, Marciano, et al.’s retrospective single-center study (n = 35 aneurysms), including procedures between March 2008 and June 2015, suggested that although the CE-MRA agreement with DSA to detect an aneurysm remnant post-stent assisted coiling treatment was better than 3D-TOF-MRA, there was no clearly statistically significant difference in accuracy. Both CE-MRA and 3D-TOF-MRA were not good at assessing the intraluminal environment of the segment of the vessel containing the FD [8]. 

Although MRA may be another option to evaluate aneurysms post-PED placement, it is an invasive procedure not suitable for all patients. In the case report by Chiu, et al., the physicians initially followed up with MRA but opted to use MRI for future follow-up studies due to the patient’s age. This case report highlights the potential pitfall of using MRIs as an imaging modality in post-PED placement follow-up of a patient with a Grade 1 subarachnoid hemorrhage secondary to a dissecting right anterior cerebral artery (ACA) aneurysm. A positive MRI finding for the reperfusion of the aneurysm led to a follow-up CTA, which demonstrated the formation of a new mid-construct occlusion. Similar to the case that we present, Chiu, et al. describe this as the first documented case to show the “reopening of a previously ‘thrombosed’ aneurysmal remnant.” Concluding remarks from this case study point toward a potential limitation of using MRI in post-PED cases, as the resolution of the central area of the stent may be obscured by artefactual resolution from the PED [9]. This case study provides concrete evidence suggesting that a single imaging modality may not be sufficient for post-PED placement follow-up.

Additional imaging strategies, such as CTA, may assist with the evaluation of aneurysm occlusion and stent patency in post-PED patients. CTA is a good imaging modality to determine stent patency and aneurysm occlusion when endosaccular coils are not present, but other noninvasive techniques, such as MRI and MRA, are better alternatives when coils are present. While MRI and MRA are good alternatives for post-PED imaging studies, as they are less affected by coil artifact, they do not provide good resolution of the device’s lumen and may incorrectly show blood flow, which can be interpreted as reperfusion [10]. Catheter angiography would certainly answer the issue definitively, but the patient refused further invasive procedures. Partially as a result of this case, we have added the four-dimensional (4D) dynamic MRA to our PED post-placement imaging protocol and plan to use this protocol at this patient's next follow-up.

## Conclusions

In summary, our case demonstrates that the use of CE-MRA for post-PED placement imaging can be misinterpreted as recanalization and may prove to be a pitfall in diagnosis. While noninvasive and noncontrast techniques are preferred when possible, we are not ready to make this transition, as there are pros and cons to each technique. Instead, we must consider the use of multiple imaging modalities in order to improve diagnostic accuracy. Our case sheds light on the importance of selecting the appropriate imaging technique(s) to evaluate aneurysms post-PED placement. Further studies are required to determine the best practice for the assessment of aneurysms post-PED placement in order to reduce the number of cases flagged as reperfusions and later deemed to be occlusions.

At the present moment, we recommend multi-imaging modalities when assessing post-PED placement. The most important guiding factor in decision-making is the patient’s willingness to endure and/or tolerance to contrast-enhanced procedures. We cannot abandon the gold standard, DSA, yet, but we also need to confirm our findings with alternate techniques, such as the CE-MRA or CTA, when appropriate. In an ideal situation, we would have liked to follow up with a catheter angiogram for our patient, but she refused additional invasive procedures. An alternate strategy is to use dynamic MRA, which does not involve contrast, to visualize flow within the PED and the aneurysm sac. The data presented in this case report is not of sufficient volume or scope to support changing one’s practice but does serve as an important reminder of the limitations of using one modality to follow these complex lesions.
